# Physiological and Biochemical Responses of *Sagittaria trifolia* L. to Phytotoxic Ethyl Acetate Fungal Extract from *Curvularia lunata* Strain CLST-01

**DOI:** 10.3390/plants12091758

**Published:** 2023-04-25

**Authors:** Kai Wang, Chang Xu, Dongyang Li, Zumin Gu

**Affiliations:** Department of Pesticide Science, Plant Protection College, Shenyang Agricultural University, Shenyang 110866, China

**Keywords:** *Sagittaria trifolia* L., *Curvularia lunata*, ethyl acetate fungal extract

## Abstract

*Curvularia lunata* (No. CLST-01), a fungal pathogen isolated from the threeleaf arrowhead (*Sagittaria trifolia* L.), has been proposed as a potential mycoherbicide for grass weeds. This paper investigated the physiological and biochemical effects of CLST-01 phytotoxic ethyl acetate fungi extract on the leaves of the threeleaf arrowhead. The results showed that the ethyl acetate fungi extract from CLST-01 can accelerate damage to the cell membrane, increase the production of malondialdehyde, and damage the cellular structure, which could decrease the number of chloroplasts after 96 h treatments. In addition, the content of chlorophyll was reduced by 49.5%, and the net photosynthetic rate, stomatal conductance, and transpiration rate were inhibited. The rates of inhibition were 90.13%, 83.74%, and 79.31%, respectively, and the intercellular CO_2_ concentration increased by 51.87% on Day 9 after treatment with a concentration of 200 μg/mL. In summary, the phytotoxic ethyl acetate fungal extract from *C. lunata* CLST-01 can inhibit the photosynthesis of the threeleaf arrowhead leaves, destroy the ultrastructure of leaves, and affect the growth of this invasive weed. Therefore, it has the potential to be developed into a mycoherbicide for weed control in crops as a natural photosynthetic inhibitor.

## 1. Introduction

The threeleaf arrowhead (*Sagittaria trifolia* L.) is a perennial aquatic plant that belongs to the *Alismataceae* (water plantains family), and is a serious weed in paddy fields in northeast China [1,2]. It drastically reduces the yield of crops by competing with cultivated crop plants for growth factors, including nutrients, water, light and space [3]. Therefore, different methods were used to control it. Mechanical methods, such as hand weeding, require huge labor and time. Threeleaf arrowheads are currently primarily managed by chemical herbicides, but in recent years, these plants have become resistant to chemical herbicides. Therefore, biological herbicides that can be used to manage the threeleaf arrowhead are urgently needed [4,5].

Biological control, particularly the use of plant pathogenic fungi to control weeds, has become an active field of weed control [6]. Plant pathogens produce active substance, which affected their plant hosts by the development of diseases [7]. Host-specific fungi have been utilized to develop bioherbicide. The fungi play a dramatic role in the physiology of their host weed plants and also produce phytotoxic compounds or other ethyl acetate fungal extracts that have activity against other weeds. Researchers have continuously recognized and discovered the fungal plant pathogen *Curvularia lunata*, and the mechanism of their interaction with plants has also gradually been identified. *C. lunata* was isolated from *Digitaria sanguinalis* and *Echinochloa crusgalli* respectively. The ability of *C. lunata* to control *D. sanguinalis* and *E. crusgalli* can reach as high as 75% and 90% in the field, respectively [8,9].

Fungal metabolites kill plants as a mechanism to destroy their cellular structures, such as mitochondria and chloroplasts, affect photosynthesis, and cause wilting, chlorosis, and even the death of infected plants [10]. *Puccinia lagenophorae* and *Albugo candida* infect host leaves and significantly decrease the prevalence of pigments, such as chl a, chl b and carotenoids [11]. Pathogens that cause foliar diseases can affect the physiology of a plant owing to the loss of healthy leaves, which has a negative impact on its ability to exchange gases and reduces the efficiency of processes related to photosynthesis [12]. Reductions in the rate of photosynthesis can also be caused by pathogens, which induce the closure of stomata and limit the availability of CO_2_ in the chloroplasts [13], which has a direct effect on the biochemical steps of photosynthesis. Photosynthetic characteristics, such as malondialdehyde (MDA) and chlorophyll a fluorescence, have been considered indicators for the photosynthetic apparatus when it is under stress, including while under infection by pathogens [14]. These changes can lead to a decrease in the photosynthetic rate, alteration to the photosynthetic apparatus, and damage to mitochondrial membranes [15]. In addition, it can decrease the number of chloroplasts. Because chloroplasts are closely related to photosynthesis, the damage of the chloroplast structure directly affects the normal function of the photosynthetic electron transport chains, and photosynthesis is weakened. Eventually, the plants appear necrotic, wilt, turn yellow, and even die. Moreover, the changes in the permeability of cell membranes and the content of MDA can damage the cell ultrastructure.

*Curvularia lunata* was a prevalent and important plant pathogenic fungus, and has the ability of degrading plants. The previous results in our research showed that the CLST-01 strain of *C. lunata*, which was isolated from the threeleaf arrowhead caused specific symptoms, such as chlorosis, necrosis and leaf spots, on plant leaves. This study aimed to understand how the ethyl acetate fungi extract of *C. lunata* CLST-01 damage the threeleaf arrowhead.

## 2. Results

### 2.1. Selection Extractant for CLST-01 Toxin

**Figure 1:**
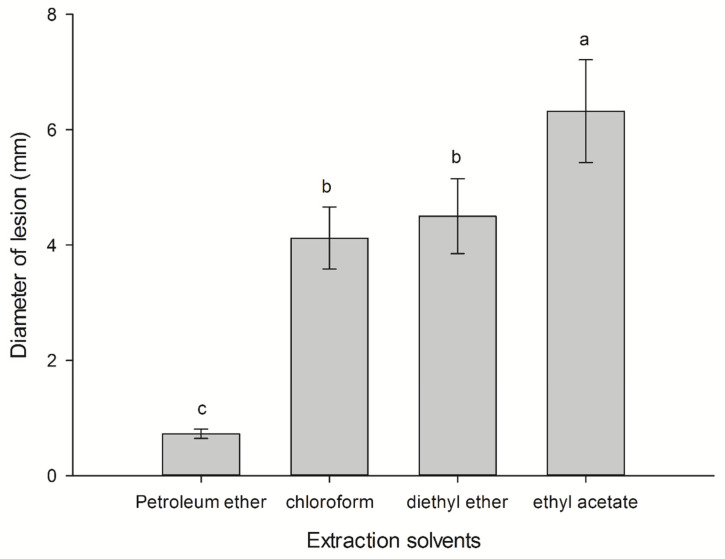
The activity of different solvents to extract phytotoxic compounds. Data are shown as mean ± standard error (*n* = 18). Different letters indicate significant differences (*p* < 0.05).

After inoculation with the pathogen, the wild arrowhead was incubated at 28 °C for 3 days, and its diameter was measured. Each treatment was repeated three times. The extraction effect of four solvents with different polarity on phytotoxic compounds in the culture filtrate is closely related to the polarity of the solvent. Among them, ethyl acetate has the best extraction effect, with 0.165 ± 0.007 g of extraction yield and a lesion diameter of 6.32 mm, which is significantly higher than that of other solvents (*p* < 0.05), so the ethyl acetate fungi extract was dissolved with 2 mL of ethyl acetate. Petroleum ether has the worst extraction effect, with almost no lesions (Figure 1). Its extraction yield was also the least, only 0.024 ± 0.0035 g. It can be seen that CLST-01 can be efficiently extracted by medium polar organic solvents, such as ethyl acetate.

### 2.2. Pathogenic Response of CLST-01

**Figure 2:**
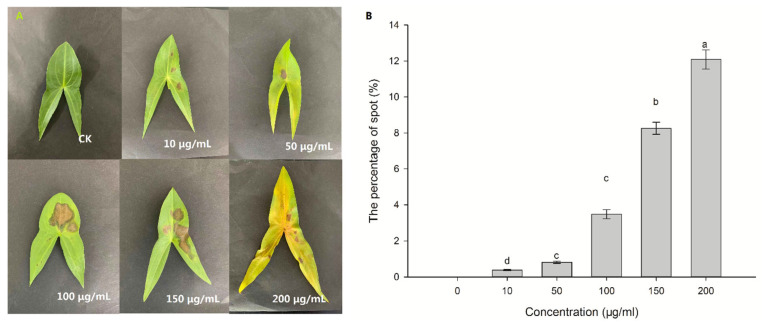
The symptomatic characteristics (**A**) and spot percentage (**B**) associated with the ethyl acetate fungal extract of CLST-01. Data are shown as mean ± standard error (*n* = 18). Different letters indicate significant differences (*p* < 0.05).

The threeleaf arrowhead leaves showed lesions after 36 h following treatment with the ethyl acetate fungi extract of CLST-01. The leaves were damaged to the greatest extent when the concentration was 200 μg/mL. The percentage of spot was 12.09% (3.69 cm^2^), which differed significantly from the other concentrations (*p* < 0.05) (Figure 2). Since the concentration was 10 μg/mL, the leaves were still subjected to the toxin, but the effect was not obvious. The area of the lesion was only 0.11 cm^2^, which was only 10% of the total leaf area.

### 2.3. Effect of CLST-01 on Chlorophyll Content

**Figure 3:**
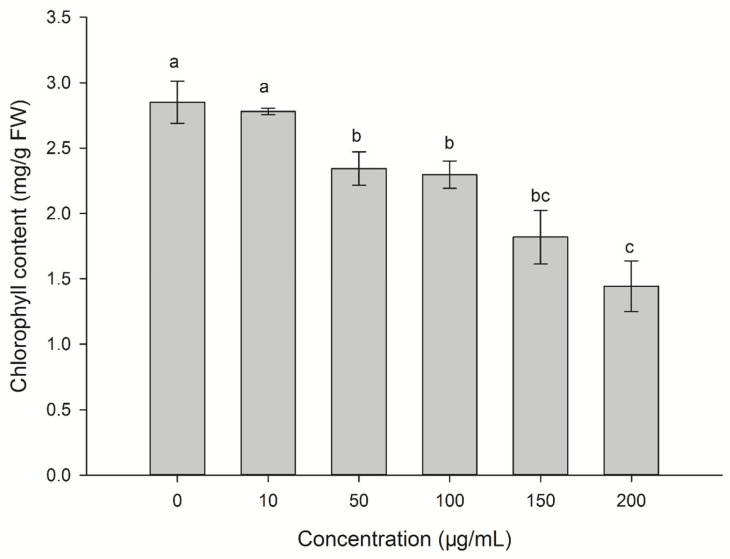
The changes of chlorophyll content in *S. trifolia* leaves associated with the ethyl acetate fungal extract of CLST-01. Data are shown as mean ± standard error (*n* = 18). Different letters indicate significant differences (*p* < 0.05).

The chlorophyll content of the threeleaf arrowhead leaves was affected after treatment with ethyl acetate fungal extract for 48 h of incubation (Figure 3). With the increase in concentration of the ethyl acetate fungal extract, the content of chlorophyll significantly decreased. At concentrations > 50 μg/mL, the chlorophyll of the leaves was significantly lower than that of the control (*p* < 0.05). As the concentrations of the ethyl acetate fungal extract increased, the content of the chlorophyll was substantially reduced by 19.3%, 19.6%, 36.1%, and 49.5%.

### 2.4. Effect of CLST-01 on Photosynthetic Rate

**Figure 4:**
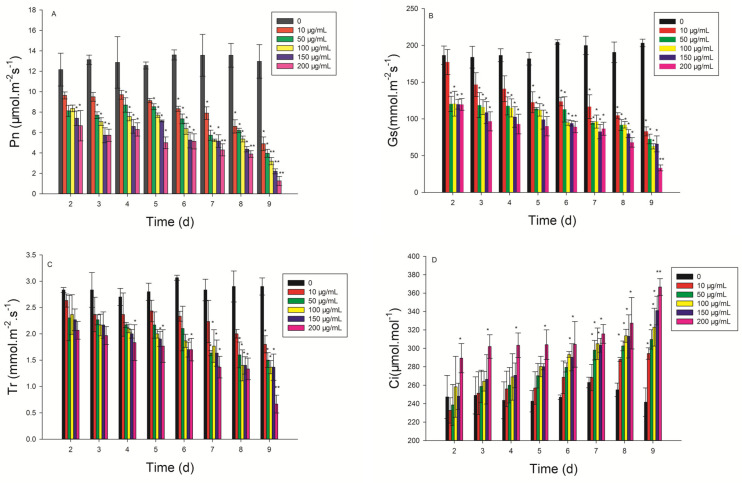
The changes of net photosynthetic rate (Pn, (**A**)), stomatal conductance (Gs, (**B**)), transpiration rate (Tr, (**C**)), and intercellular CO_2_ concentration (Ci, (**D**)) in *S. trifolia* leaves associated with the metabolite of CLST-01. Data are shown as mean ± standard error (*n* = 18). ** *p* < 0.01, * *p* < 0.05.

The photosynthetic parameters changed noticeably as the concentration increased (Figure 4). From the second day of the treatment with ethyl acetate fungal extract, the net photosynthetic rate (Pn) and stomatal conductance (Gs) of the leaves decreased significantly (*p* < 0.05) compared with the control at 150 μg/mL and 200 μg/mL, respectively (Figure 4A–C). The Pn, Gs, and Tr decreased by 90.13%, 83.74%, and 79.31% at the 200 μg/mL concentration of ethyl acetate fungal extract after 9 days of treatment, respectively. The Ci increased significantly at 200 μg/mL (*p* ≤ 0.05) during the 9 days of treatment (Figure 4D). After treatment with 200 μg/mL ethyl acetate fungal extract for 9 days, the Ci increased by 51.87%, leading to the photosynthetic dysfunction of the leaves.

### 2.5. Effect of CLST-01 on Malondialdehyd

**Figure 5:**
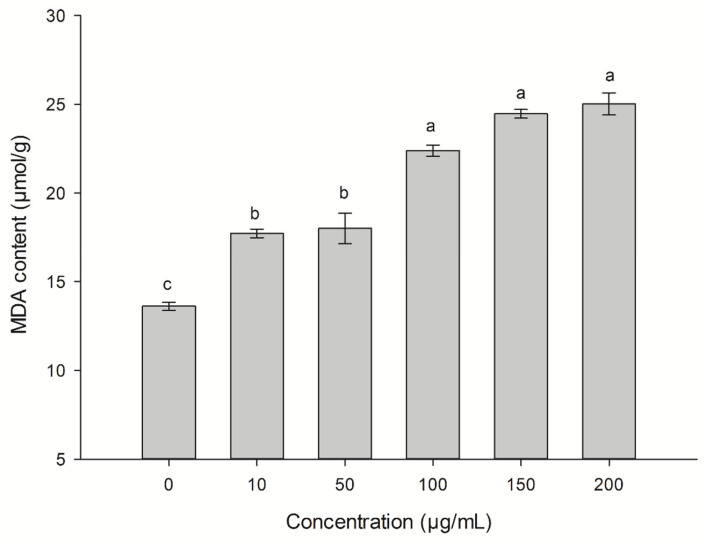
The changes of malondialdehyde (MDA) content in *S. trifolia* leaves associated with the ethyl acetate fungal extract of CLST-01. Data are shown as mean ± standard error (*n* = 18). Different letters indicate significant differences (*p* < 0.05).

The MDA in the threeleaf arrowhead leaves was significantly higher (*p* ≤ 0.05) than that of the control with 10 μg/mL, 50 μg/mL, 100 μg/mL, 150 μg/mL, and 200 μg/mL, leading to increases of 30.17%, 32.23%, 64.47%, 79.67%, and 83.76%, respectively (Figure 5). As the concentration increased, the content of MDA also increased, but there was no significant difference between the concentrations of 100 μg/mL, 150 μg/mL, and 200 μg/mL.

### 2.6. Effect of CLST-01 on Cell Membrane Permeability

**Figure 6:**
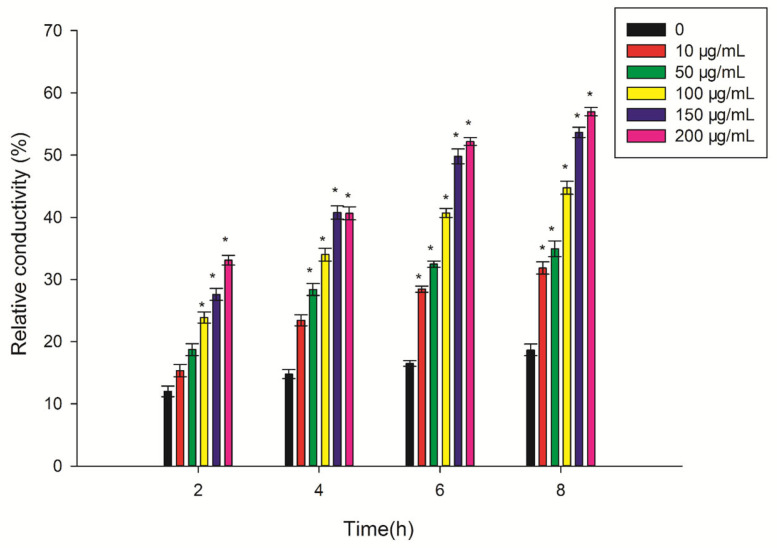
The changes of relative conductivity in *S. trifolia* leaves associated with the ethyl acetate fungal extract of CLST-01. Data are shown as mean ± standard error (*n* = 18). * *p* < 0.05.

As shown in Figure 6 treatment with the ethyl acetate fungal extract of CLST-01 had an impact on the cell membrane permeability of the leaves. The relative conductivity of the extract increased with the increase in the treatment time using the same concentration. The relative conductivity was significantly higher (*p* ≤ 0.05) than that of the control following treatment with 100 μg/mL, 150 μg/mL, and 200 μg/mL. The relative conductivities reached a peak value at 8 h and increased by 70.56%, 87.25%, 139.7%, 187.4%, and 205.3%, respectively.

### 2.7. TEM

**Figure 7:**
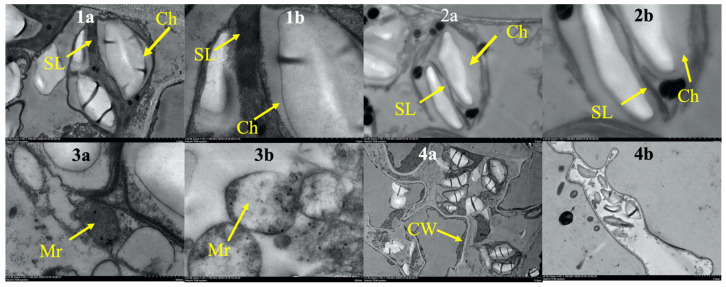
The effect of the ethyl acetate fungal extract from CLST-01 on the leaf ultrastructure of *Sagittaria trifoli*. (**1a**) and (**1b**) are the intracellular chloroplast structure after 96 h of sterile water treatment, (**2a**) and (**2b**) are the intracellular chloroplast structure after 48 h of crude toxin treatment, (**3a**) is the intracellular mitochondrial structure after 96 h of sterile water treatment, (**3b**) is the intracellular mitochondrial structure after 48 h of crude toxin treatment, (**4a**) is the cell structure after 96 h of sterile water treatment, (**4b**) is the cell structure after 96 h of crude toxin treatment. CW: Cell wall; Ch: Chloroplast; SL: Stroma lamella; Mr: Mitochondrion.

The ultrastructure of leaves was significantly affected by the ethyl acetate fungal extract. The ultrastructure of the control chloroplasts was regular, and the chloroplast lamellae were complete and orderly. There were fewer osmiophilic granules in the chloroplasts. The mitochondrial double-layer membrane structure was complete, and the inner cristae were clearly visible (Figure 7(1a,1b,3a,4a)). After 48 h of treatment, the number of chloroplasts decreased. The lamellae were disorderly, and the double-layer membrane structure had been destroyed. Osmiophilic granules became larger and more frequent. A few mitochondria still had inner cristae, but the membrane structure was seriously damaged, and some mitochondrial outer membranes disappeared (Figure 7(2a,2b)). After 96 h of treatment, the structure of cells disintegrated. The organelles were completely disintegrated, and the chloroplasts and mitochondria disintegrated into flocs and dissolved in the cytoplasm (Figure 7(3b,4b)).

## 3. Discussion

Pesticides are used to increase the yields of crops, but the unreasonable use of chemical pesticides leads to environmental pollution and weeds that are resistant to these chemicals. Chemically synthesized pesticides are not allowed in organic agriculture; thus, biological pesticides have become the focus of intensive research in recent years [16,17]. Mycoherbicides are an important means to control phytopathogenic fungi because they can be specific for weeds, safe for the environment, and often cost effective [18,19,20]. Fungi can exert herbicidal effects primarily owing to the role of their metabolites. It is particularly important to study the mechanism of metabolites [21].

Extracting metabolites with herbicidal activity from fungi can help to develop natural herbicides, evaluate their bioactivities, and screen metabolites with higher herbicidal activity [22]. The results of bioactivities showed that a higher concentration of CLST-01 ethyl acetate fungal extract was more toxic to the threeleaf arrowhead leaves, resulting in more serious chlorosis (Figure 1). This indicated that the extracted metabolites are toxic to the threeleaf arrowhead leaves. Previous studies also showed that as the concentration of metabolites increases, phytotoxic responses will be more serious [23,24]. When plant pathogenic fungi interact with their hosts, they often produce metabolites that can destroy the physiological and biochemical functions and decrease the content of chlorophyll, which leads to the withering, yellowing, and even the eventual death of the host plants [25].

Chlorophyll plays a vital role in plant photosynthesis [26]. Plant leaves affected by phytotoxic compounds appear chlorotic, and the chlorophyll content will inevitably decrease, indicating that the ethyl acetate fungal extract of CLST-01 affects the biosynthetic pathway of chlorophyll or damages its structure [27]. It is also related to the active sites of the toxin, which primarily include the plasma membrane, chloroplast, mitochondria, and defensive enzymes. Research found that susceptible rice-*Rhizoctonia solani* interactions were found to result in the structural disintegration and distortion of the chloroplast grana [28]. Treating host plants with some substances caused the chloroplast envelope to dissolve and disappear with degraded layers, leakage of the chloroplast contents, as well as swollen-shaped chloroplasts in the leaf center; thus, the physiological activities of the cells were severely inhibited [29,30]. The results of this study are consistent with the ones described above (Figure 6). The content of the chlorophyll in the threeleaf arrowhead leaves was affected after 48 h of incubation with the ethyl acetate fungal extract (Figure 2). Chlorophyll may lead to the weakening of photosynthesis of plants and even result in the death of plant. The results of an ultrastructural analysis of threeleaf arrowhead leaves indicated that the content of chlorophyll in their leaves decreases, and the chloroplasts and mitochondria gradually disintegrate. This indicates that the ethyl acetate fungal extract had a damaging effect on the ultrastructure of the plants and eventually inhibited the synthesis of chlorophyll.

The damage to the cellular structure is related to MDA and cell membrane permeability [31]. The ethyl acetate fungal extract of *C. lunata* can accelerate the damage to cell membranes in threeleaf arrowhead leaves (Figure 5), resulting increase the production of MDA (Figure 4), and the damage to structure of the cell (Figure 6). In addition to the effects of to Pn, Gs, Tr, and Ci on the content of chlorophyll, they also affect photosynthesis. Photosynthesis is inhibited when the plants are subjected to stress [5]. Faith found that the rates of photosynthetic were reduced in beans (*Phaseolus vulgaris*) infected with white mold (*Sclerotinia sclerotiorum*), but the stomatal conductance did not vary [32] Positive and direct associations were found between Pn and Gs [33]. This research also confirmed that the photosynthesis of beans is inhibited by the ethyl acetate fungal extract of CLST-01. Studies found that aubergine (*Solanum melongena*) plants infected with *Verticillium dahlia* had limited Gs and limitations to the mesophyll owing to an increase in the Ci [34]. Eucalyptus infected with *Ceratocystis* blight (*Ceratocystis fimbriata*) indicated that Gs and Tr were significantly reduced. These results suggest that *C*. *fimbriata* can cause the partial closure of the stomata to prevent water loss and a consequent reduction in photosynthesis and the Tr, which, in turn, leads to a decrease in the growth of the plant [35]. In this research, the Pn, Tr, and Gs of threeleaf arrowhead leaves decreased by 90.13%, 83.74%, and 79.31%, respectively, and Ci significantly increased by 51.87%. The photosynthesis becomes disordered and the plants are damaged (Figure 3).

Previous studies have shown that multiple metabolites can be obtained from the filtrate of *Curvularia lunata*. Macri et al., separated two small molecules from the filtrate through organic solvent extraction and gel column chromatography, one of which has a molecular weight of about 350 [36]. Xiao et al., found that the main components of the metabolites of *Curvularia* are proteins and soluble sugars [37]. Liu et al., isolated a compound (C_7_O_4_H_8_) from the ethyl acetate extract of *Curvularia lunata*, and its structure was identified as methyl 5-(hydroxymethyl) furan-2-carboxylate [38].

Studies have shown that small molecule substances isolated from culture filtrate can produce necrotic spots on maize leaves, causing cell electrolyte leakage [36]. Based on the results of this study, it is speculated that the metabolites of *Curvularia lunata* can also cause lipid peroxidation, disrupting the integrity of cell membranes. In the experiment, it was also found that the extract of ethyl acetate can reduce chlorophyll content and affect photosynthetic parameters, indicating that the target of action may serve as a pigment synthesis inhibitor. By inhibiting the synthesis of chlorophyll on the thylakoid membrane, the photosynthetic system is damaged, leading to plant death.

The experiment clearly demonstrated that the ethyl acetate fungal extract from pathogens causing foliar diseases affects the physiology of a plant by losing healthy leaf area, which has a negative impact on its ability to exchange gases or reduces the efficiency of photosynthesis-related processes. The bioactive ethyl acetate fungal extract from the fungi maybe become a new source of finding lead compounds with herbicidal activities.

## 4. Materials and Methods

### 4.1. Plant Materials

Threeleaf arrowhead corms were collected in October 2021 and stored at 4 °C. Approximately 3–4 corms were planted in 3 cm deep soil in plastic pots with a diameter of 25 cm. Plastic pots were placed in a greenhouse at 25 ± 5 °C during the day and 15 ± 5 °C at night. The photoperiod was 12 h light and 12 h dark.

### 4.2. Fungal Species and Extraction

The fungus was originally isolated from naturally diseased threeleaf arrowhead leaves, identified as *C. lunata* and designated CLST-01. The fungus kept at 4 °C was routinely grown on potato dextrose agar (PDA) plates or slants and it was used fresh for the experiments. *C*. *lunata* strains were grown on potato dextrose agar (PDA) for 5 d at 28 °C in the dark. The mycelial disks (Φ = 7.5 mm) were obtained from the edge of the colony. Erlenmeyer flasks (250 mL) that contained 100 mL PSK were sterilized for 30 min at 121 °C. After cooling, each flask was inoculated with three mycelial disks prepared as described above; then, they were kept in an orbiting shaker in the dark at 28 °C, 150 rpm for 14 days. After incubation, the cultured mixture was filtered through four layers of cheesecloth and centrifuged for 20 min at 15,000 rpm using a centrifuge.

In order to optimize the extracting conditions and improve the extraction effect. The supernatant was extracted three times with an equal volume of petroleum ether, chloroform, ether, and ethyl acetate. The extracts were combined and then evaporated in a 50 °C water bath to obtain the yellow-brown ethyl acetate fungal extract. The biological activity of extracted toxin was determined by the acupuncture inoculation method. The experiment was repeated three times.

### 4.3. Phytotoxicity Test

The in vitro leaf acupuncture method involved selecting Stage 3–4 seedlings of threeleaf arrowheads, washing the surface dust off with tap water, and rinsing with distilled water for 3 s. After they had air-dried, the leaves were injected with a micro syringe to cause slight damage, but the leaves were not stabbed. A volume of 0.5 mL solution of ethyl acetate fungal extract (10, 50, 100, 150, and 200 μg/mL) was dropped onto the wound. After sucking up the drops of ethyl acetate fungal extract, the leaf was placed in an enamel dish covered with sterile gauze, cultured at 28 °C in an incubator with a relative humidity of 40% ± 2%. The diameter of the lesions was measured after 4 days using sterile water as the control. The treatment with 10 *Sagittaria trifolia* was repeated three times (*n* = 3).

### 4.4. Chlorophyll Content

The content of chlorophyll was determined as described by Lu et al. [39]. The experiment with 10 *Sagittaria trifolia* was repeated three times (*n* = 3).

The second and third leaves were chosen from the bottom of the same growing and healthy threeleaf arrowhead. The leaves were rinsed with running water for 10 min and then washed three times with distilled water. One leaf was placed in a Petri dish (Φ 9 cm) that contained 10 mL of different concentrations of ethyl acetate fungal extract (10, 50, 100, 150, and 200 μg/mL) and sterile distilled water as a control. The leaves were removed after incubation under light at 25 °C for 48 h and placed in a tube that contained 10 mL of extraction solvent (acetone: ethanol: distilled water = 4.5:4.5:1). A volume of 2 mL polyvinylpyrrolidone (PVP) was added and extracted at 25 °C in the dark for 24 h. The supernatant and the extraction were a solvent control, and the absorbance at 645 and 663 nm was measured using a UV-Vis spectrophotometer. Each treatment was conducted in triplicate. The content of chlorophyll was calculated using the following formula:

$$\mathrm{Chlorophyll}\;\mathrm{content}\;\left(\mathrm{mg}/g\right)=\frac{\left(20{.29\;\times \;\mathrm{OD}}_{645}+8{.05\;\times \;\mathrm{OD}}_{663}\right)\;\times \;V}{1000\;\times \;W}$$

where *V* is the volume of the extraction (mL) and *W* is the fresh weight of the sample (g).

### 4.5. Photosynthetic Rate

The second and third pairs of leaves were selected from the bottom of the same growing healthy plants. The front and back sides of the leaves were wiped with 75% alcohol and then with sterile water. Sterile gauze was used to absorb the water on the surface of the leaves, and then a solution of metabolite (10, 50, 100, 150 or 200 μg/mL) was sprayed at the same time each day for 8 days. The net photosynthetic rate (Pn, μmol·m^−2^·s^−1^), stomatal conductance (Gs, mmol·m^−2^·s^−1^), transpiration rate (Tr, mmol·m^−2^·s^−1^), and intercellular CO_2_ concentration Ci (μmolmol^−1^) were measured using a Ciras-3 portable photosynthesis analyser (PPSystems, Amesbury, MA, USA) from 9:30 to 11:00 AM from Day 2 to Day 9. The light intensity was 600 μmol·m^−2^·s^−1^. The experimental environment temperature was controlled at 25 ± 1 °C with a relative humidity of 40% ± 2%; a treatment of sterile water was used as the control, and each treatment with 10 *Sagittaria trifolia* was conducted in triplicate [40].

### 4.6. Cell Membrane Permeability

The second and third pairs of leaves were selected from the bottom of the same growing healthy threeleaf arrowhead plants. The front and back sides of the leaves were wiped with 75% alcohol followed with sterile water. Sterile gauze was used to absorb the water on the surface of the leaves. The leaves were cut into 0.5 cm × 0.5 cm pieces using a double blade and avoiding the main vein. A total of 0.5 g of leaf cubes were weighed and placed in test tubes that contained 10, 50, 100, 150, and 200 μg/mL extraction solution of CLST-01, respectively. Each treatment with 10 *Sagittaria trifolia* was conducted in triplicate. After vacuum decompression for 10 min, the leaves had completely sunk to the bottom of the tubes and were incubated at 25 °C for 2, 4, 6, and 8 h. The electrical conductivity of the leaf tissue extracts of the threeleaf arrowroot was measured using a djs-1c conductivity meter (Mettler Toledo, Columbus, OH, USA) at room temperature. At last, the treatment groups were boiled for 20 min, and the maximum electrical conductivity of the leaf tissue extract was measured after cooling to room temperature. The relative electrical conductivity of the crude toxin to leaf tissue cells was calculated as follows [29]:

$$\mathrm{Relative}\;\mathrm{conductivity}\;\left(\%\right)=\frac{\mathrm{Processed}\;\mathrm{conductivity}}{\mathrm{Maximum}\;\mathrm{conductivity}}\times 100$$

### 4.7. Malondialdehyde (MDA) Content

The blade treatment method was the same as that used in Section 2.6. After treatment with distilled water 25 °C for 4 h, the samples were taken out, rinsed three times with distilled water, and wiped dry to indicate moisture. A volume of 5 mL PBS (pH 7.8, 1% PVP) and a small amount of quartz sand were added, ground in an ice bath, and centrifuged for 30 min at 4 °C, 12,000 r·min^−1^. A volume of 2 mL of supernatant was mixed with 2 mL of thiobarbituric acid (TBA) (20% trichloroacetic acid ([TCA], 0.5% TBA), cooled to room temperature for 4 °C, and centrifuged for 10 min at 12,000 r·min^−1^. Each treatment with 10 *Sagittaria trifolia* was conducted in triplicate The absorbance values A_450_, A_532_, and A_600_ of the supernatant were measured by spectrophotometry, and the MDA content was calculated using the following formula [8]:

MDA content (μmol/g) = [6.45 × (A_532_ − A_600_) − 0.56 × A_450_] × Extract × 1000/*W*

where *W* is the sample fresh weight (g).

### 4.8. Transmission Electron Microscopy (TEM)

Healthy leaves were treated with 50 μg/mL of ethyl acetate fungal extract for 48 h and 96 h, and the ultrastructure of the leaves was observed. The leaf fragments (1 mm × 3 mm) were quickly placed in a fixed penicillin bottle that contained 2.5% (*v/v*) glutaraldehyde and was emptied with a syringe until the leaves sank to the bottom. The sample was then transferred to 4 °C for at least 24 h. The samples were post-fixed with 1% (*v/v*) osmium tetroxide at 25 °C for 2 h. After that, the stationary solution was dehydrated using an ethanol solution (30% to 100%, *v/v*) in the gradient, and each time it was incubated to permeate for 15–30 min and embedded into spi-812 resin. The sample was sliced and stained with uranyl acetate and lead citrate (EM UC7; Leica, Wetzlar, Germany). The samples were observed by TEM (ht7700; Hitachi, Tokyo, Japan) [34]. Each time point was repeated six times.

### 4.9. Statistical Analysis

At least three independent replicates were used in all the experiments. All the data were expressed as the mean ± SE by measuring three independent replicates, and statistically analyzed using an analysis of variance (ANOVA). The mean separations were conducted using Duncan’s multiple range tests. The different small letters in the tables and figures (a, b, c, and d) indicate the significant differences of the parameter value at *p* ≤ 0.05 using SPSS 21.0 (IBM, Inc., Armonk, NY, USA).

## 5. Conclusions

This study indicates that the ethyl acetate fungal extract of *Curvularia lunata* CLST-01 can have a strong pathogenic effect on threeleaf arrowheads. It can accelerate the damage to cell membranes and the production of MDA, which can damage the cellular structure. In addition, it could decrease the number of chloroplasts, inhibit photosynthesis, and finally, cause the death of leaves owing to chlorosis.

## Data Availability

Not applicable.
